# Effects of an Herbal Source of Choline on Diet Digestibility and Palatability, Blood Lipid Profile, Liver Morphology, and Cardiac Function in Dogs

**DOI:** 10.3390/ani12192658

**Published:** 2022-10-03

**Authors:** Rosandra Colpani do Nascimento, Camilla Mariane Menezes Souza, Taís Silvino Bastos, Gislaine Cristina Bill Kaelle, Simone Gisele de Oliveira, Ananda Portella Félix

**Affiliations:** Department of Animal Science, Sector of Agricultural Sciences, Federal University of Paraná, 1540 Funcionários Street, Curitiba 80035-050, PR, Brazil

**Keywords:** blood metabolites, canine, choline chloride, herbal additive, liver morphology, lipidic metabolism

## Abstract

**Simple Summary:**

Choline is an essential nutrient for dogs, and its dietary supplementation is usually made using choline chloride. However, this compound is highly hygroscopic—meaning it absorbs moisture from the air—which makes it difficult to manipulate during animal food production. Stable herbal additives rich in phosphatidylcholine may be used as an alternative source of choline. In light of this, this study compared the effects of an herbal source of choline with choline chloride on diet digestibility and palatability, blood metabolites, liver morphology, and cardiac function in adult dogs. Our results suggest that the herbal source of choline is a possible substitute for choline chloride in dog diets, in addition to reducing the activity of liver enzymes, total cholesterol, and serum triglycerides. This study is relevant to the pet food market since it concerns pet owners interested in natural alternative foods.

**Abstract:**

We aimed to evaluate the effects of an herbal source of choline on the coefficients of total tract apparent digestibility (CTTAD), diet palatability, fecal characteristics, blood variables, liver morphology, and cardiac function of dogs. Sixteen adult dogs were randomly assigned to two groups (*n* = 8) which were fed two different diets for 45 days: control, containing 0.28% choline chloride 60, and test, containing 0.14% of an herbal source of choline. Feces were collected between days 39 and 44 to determine nutrient CTTAD and fecal characteristics. On days 0 and 45, blood samples were collected and the liver morphology was evaluated. Cardiac function, in turn, was evaluated only on day 45, and the palatability test was performed on two consecutive days (*n* = 32). There were no changes in nutrient CTTAD, diet palatability, or fecal characteristics of dogs fed the test diet (*p* > 0.05). However, on day 45, dogs fed the test diet showed lower (*p* < 0.05) serum total cholesterol, triglycerides, alkaline phosphatase, and alanine aminotransferase when compared to the control group. We concluded that the herbal source of choline can be a possible substitute for choline chloride in dog nutrition.

## 1. Introduction

Choline is an essential nutrient involved in the structure and metabolism of phospholipids (phosphatidylcholine and lecithin), as well as a precursor of acetylcholine, an important neurotransmitter [1]. Furthermore, it plays a crucial role in lipid metabolism, especially in the liver, since it is a lipotropic factor for preventing the abnormal accumulation of fatty acids in liver tissue (hepatic steatosis) [2].

Choline requirements for adult dogs range from 1640 to 1890 mg/1000 g of dietary dry matter [3]. Usually, this nutrient is supplemented in dogs’ diet through choline chloride. However, some of its properties, such as high hygroscopicity and corrosiveness, limit its practical use [4]. Consequently, it is difficult to manipulate during animal food or premix production, impairing its premixing with other micro-ingredients and causing the loss of vitamins [5], making the study of alternative sources of choline for pet food necessary.

The plants *Trachyspermum ammi*, *Citrullus colocynthis*, *Achyranthes aspera*, and *Azadirachta indica* present the conjugated form of choline—with high concentrations of phosphatidylcholine—and are potential alternative sources to the chloride [4]. In addition to not having the same undesirable properties as choline chloride, these herbal additives have functional metabolites that benefit animals’ health [5,6]. Furthermore, they can be used in organic foods or in those with a ‘natural’ advertising appeal [7,8]. On the other hand, they have volatile aromatic compounds [9,10] that might affect diet palatability [11].

Studies with domestic species, such as poultry and cattle, have already corroborated the use of herbal sources of choline in these animals’ nutrition [4,8,12]. The recommended bioequivalence by these studies is one unit of herbal source for every 2.52 units of choline from chloride [4]. However, we found only two studies in dogs, and they originate from the same experiment. In these studies, six breeds were used (Beagle, Schnauzer, Bichon Frise, Dachshund, Airedale Terrier, and Jack Russell) for around 17 to 60 days. They recommend one unit of herbal source (400 mg/kg of diet) for 5 units of choline (2000 mg/kg of diet) from chloride [5,11]. Nevertheless, the authors observed that higher dietary concentrations of choline herbal source (800 mg/kg) resulted in additional beneficial effects on the body, such as modulation of gene expression of factors related to cardiovascular and metabolic disease prevention, without causing any adverse result [5].

In light of this, the present study aimed to evaluate the effects of an herbal source of choline on the coefficients of total tract apparent digestibility (CTTAD), diet palatability, fecal characteristics, blood variables, and hepatic and cardiac functions of dogs.

## 2. Materials and Methods

All animal procedures were approved by the Ethics Committee on Animal Use (CEUA) of the Agricultural Sciences Sector of the Federal University of Paraná, Curitiba, state of Paraná, Brazil (protocol No. 023/2019).

### 2.1. Animals and Facilities

The study lasted 45 days and was carried out in the Laboratory of Studies on Canine Nutrition, located in Curitiba, state of Paraná, Brazil (25°25′40″ S, 49°16′23″ W, and an average altitude of 934 m).

Sixteen adult beagle dogs (*Canis lupus familiaris*) (eight males and eight females), with a mean age of 4.5 ± 0.1 years and mean body weight of 13.68 ± 2.20 kg, were distributed in a completely randomized design. The animals underwent clinical and physical examination before and after the experimental period and had a mean body condition score of 4.6 ± 0.1 on a scale of 1 to 9 (1 = thin and 9 = obese; scores of 4 and 5 are considered as ideal), according to Laflamme [13].

During most of the diet adaptation period, the dogs had supervised free access to an outdoor area of 1137.84 m^2^ for 4 h/day for both voluntary exercise and socialization with other dogs and laboratory personnel. During the fecal collection period, they were housed individually in covered masonry stalls (5 m long × 2 m wide) with bars on the side walls, allowing visual contact and limited interaction with neighboring dogs. The animals also had access to a bed and water ad libitum throughout the experiment. The ambient temperature ranged from 16 °C to 28 °C with a 12 h light-dark cycle (light from 6 am to 6 pm).

### 2.2. Experimental Diets

Two diets were evaluated: control, containing 0.28% of choline chloride 60 (minimum 52% choline according to the fabricant), and test, containing 0.14% of the herbal source of choline (Biocholine Powder^®^, NutriQuest Nutrição Animal LTDA, Campinas, State of São Paulo, Brazil). The herbal source of choline contained a minimum of 1.6% phosphatidylcholine and was extracted from the following plants: *Trachyspermum amni*, *Citrullus colocynthis*, *Achyranthus aspera*, and *Azadirachta indica*. The amount of added choline chloride provided approximately 1456 mg of choline/kg of diet, which, together with the composition of the other ingredients, resulted in a calculated amount of 2221 mg of choline/kg of control diet. This amount exceeds the minimum 1890 mg of choline/kg of diet recommended by FEDIAF [3], considering an intake of 95 kcal/kg of body weight^0.75^/day and a 4000 kcal/kg diet. The test diet presented a calculated amount of choline of 765 mg/kg, considering the amount from the ingredients plus phosphatidylcholine from the herbal source. Bioequivalence of the herbal source of choline was considered close to 1:1 in relation to the amount of synthetic choline added to the diet (1400 mg of herbal choline: 1456 mg of choline from choline chloride/kg of diet).

A basal diet was formulated to meet the needs of adult dogs in maintenance, according to the FEDIAF [3], from which the two experimental diets were formed, differing only in the source of choline inclusion. The diet ingredients were ground in 1 mm sieves and extruded in a single screw extruder. The diet contained the following ingredients in its composition: poultry offal meal, porcine protein isolate, meat and bone meal, rice grits, rice bran, wheat bran, corn, corn gluten meal 60, flax seeds, zeolite, inulin, potassium chloride, cellulose, poultry oil, chicken and pork liver hydrolysate, antifungal propionic acid, BHT and BHA antioxidants, sodium chloride, yucca extract, lysine, DL-methionine, salmon oil, beet pulp; and vitamin–mineral premix with no choline source: folic acid (0.80 mg), pantothenic acid (16 mg), biotin (0.3 mg), vitamin A (20,000 IU), vitamin B1 (4 mg), vitamin B2 (8 mg), vitamin B12 (32 ug), vitamin B6 (4.8 mg), niacin (56 mg), vitamin D3 (2000 IU), vitamin K3 (4.8 mg), vitamin C (350 mg), copper (15 mg), iron (100 mg), zinc (150 mg), iodine (1.5 mg), manganese (30 mg), and selenium (0.20 mg). The chemical composition of the experimental diets is shown in Table 1.

### 2.3. Digestibility and Fecal Characteristics

The digestibility assay followed the recommendations of the AAFCO [14], with an adaptation period (39 days) followed by a phase of total feces collection (5 days). The animals were fed twice a day (at 8:30 am and 4:30 pm) with a sufficient amount of food to meet their metabolizable energy needs (MEN, also referred to as ME), according to the equation proposed by the NRC [15]: kcal/day = 95–130 × weight (kg)^0.75^. They were weighed weekly and feed amounts were adjusted for weight maintenance.

During the total collection period, the feces were collected at least twice a day, weighed, and frozen (−14 °C) in identified individual containers, constituting a composite of feces from each animal. At the end of this period, they were thawed, homogenized separately, forming a composite sample from each animal, and dried in a forced-ventilation oven at 55 °C until constant weight. Subsequently, dried feces and food were ground separately in a Willey hammer mill (Arthur H. Thomas Co., Philadelphia, PA, USA) with 1 mm sieves.

Both feces and diets were analyzed to determine dry matter (DM) at 105 °C for 12 h, acid-hydrolyzed ether extract (AEE, method 954.02), and ash contents (method 942.05), according to the AOAC [16]. Nitrogen (N) was analyzed using method 954.01 and crude protein (CP) was calculated as N × 6.25 [16]. Organic matter (OM) was calculated as 100—ash percentage. Gross energy (GE) was determined using a bomb calorimeter (Parr Instrument Co., Model 1261, Moline, IL, USA). Coefficients of total tract apparent digestibility (CTTAD) and ME were estimated according to AAFCO [14] and based on the following equations:

CTTAD (%) = [(g nutrient ingested − g nutrient excreted)/g nutrient ingested] × 100.

ME (kcal·g−1) = {kcal·g−1 GE ingested − kcal·g−1 GE excreted in feces − [(g CP ingested − g CP excreted in feces) × 1.25 kcal·g−1]}/g of food ingested.

Fecal characteristics were evaluated at the end of the experimental period (day 45) by analyzing total fecal dry matter (DMf content) and fecal consistency (determined by fecal score) [17]. The fecal score was always evaluated by the same researcher, who assigned scores from 1 to 5, as follows: 1 = pasty, unformed feces; 2 = soft and malformed feces; 3 = soft, formed, and moist feces; 4 = well-formed and consistent feces; 5 = well-formed, hard, and dry feces. Fecal pH was analyzed using a digital pHmeter (331, Politeste Instrumentos de Teste Ltd.a, São Paulo, State of São Paulo, Brazil), according to Kaelle et al. [18].

### 2.4. Blood Analysis

On days 0 and 45 of the experiment, preprandial blood samples were collected from all animals through jugular puncture after 12-h fasting. The collected samples consisted of 3 mL of blood with EDTA anticoagulant for complete blood count analysis, and 3 mL of blood with no anticoagulant for serum analysis. The samples were then transported under refrigeration (4 °C) for immediate processing in the laboratory. The following variables were evaluated using the semiautomatic method CC—530/Microscopy: number and concentration of red blood cells and hemoglobin, total leukocytes, albumin, globulin, plasma proteins, and total proteins. The activities of alanine aminotransferase (ALT), alkaline phosphatase (ALP), and gamma-glutamyl transpeptidase (GGT) enzymes, as well as serum levels of total cholesterol, LDL, HDL, and triglycerides, were also evaluated using the automated colorimetric method of the Cobas Mira Plus Chemistry System^®^.

We used a Dialab^®^ commercial chemical kit and BS-200 chemical analysis equipment (Mindray Chemistry Analyzer^®^, Shenzhen, China) for the measurement of total proteins and albumin. Hematological analyses were performed using BC 2800 vet equipment (Mindray Auto-Hematologic Analyzer^®^). Coverslips for cell counting were stained using the Rapid Panoptic method. The globulin count determination was estimated by the difference between total protein count and albumin.

### 2.5. Ultrasonography of the Abdominal Region

The ultrasound examination was performed on days 0 and 45 by the same experienced examiner using a model Logic32 ultrasound equipment and linear (6 to 10 MHz) and convex (2 to 5 MHz) multifrequency transducers. A trichotomy in the ventral abdominal region was performed before the examination. The dogs were physically restrained and positioned in right lateral, left lateral, or dorsal decubitus for evaluation of the liver parenchyma in sagittal/parasagittal and transverse sections. The metrics used for the assessment of liver size ranged from 0 to 2, where 0 = normal, 1 = enlarged (fatty infiltration/hepatopathy), and 2 = considerably enlarged (hepatopathy); for the splenic size, we considered 0 = normal, 1 = enlarged (reactional), and 2 = considerably enlarged (congestion/reactional); and for parenchymal organs echogenicity, 0 = normal, 1 = echogenic (clearer), and 2 = hyperechogenic (tissue fibrosis).

### 2.6. Echocardiogram and Blood Pressure

Echocardiography and blood pressure were measured on day 45 by an experienced professional using a Phillips Affinit 50 cardiac evaluation machine with a 3–8 MHz transducer. Firstly, a trichotomy was performed in the metacarpal palmar region, near the pad, where the pulse was palpable. A vascular Doppler device (Microem Ltd.a, Ribeirão Preto, state of São Paulo, Brazil) was used for blood pressure measurement with the animals positioned in right lateral decubitus. For the echocardiogram, the dogs were positioned both in right and left lateral decubitus with attached electrocardiogram pads, according to the recommendations of the American College of Veterinary Internal Medicine [19].

### 2.7. Palatability

The palatability test was performed on the two days following the end of the experiment. During its realization, the dogs were kept individually locked in the kennels for approximately 30 min.

Both control (choline chloride) and test (herbal source) diets were fed in amounts 30% higher than the NRC [15] recommendations for ME requirements for dogs in maintenance. Palatability was then determined by the animals’ first choice among the offered diets and their intake ratio. Diets were given once a day at 8:30 am for 30 min. Subsequently, the amounts of diet provided and the respective leftovers were quantified to calculate the intake ratio. Both bowls were removed when a diet was completely consumed before the end of the 30 min period. The first choice was considered as the first bowl approached by the animal during the simultaneous food offering, according to [20]. The bowls’ position was alternated on the second day of testing to avoid positional preferences.

Intake ratio was calculated based on the relative intake (supplied—leftovers) of the diets (A and B), in which:

Intake ratio = [ingested g of diet A or B/total g consumed (A + B)]

### 2.8. Statistical Analysis

All data were previously submitted to the Shapiro-Wilk normality test and Bartlett’s test for homogeneity of variances. Data with normal distributions were analyzed by the Student’s *t*-test at a 5% significance level. Non-parametric data were analyzed by Wilcoxon’s test (*p* < 0.05).

Blood data were analyzed considering a completely randomized design in a split-plot arrangement (plots = diets and subplots = initial and final periods), totaling eight repetitions per treatment. Normally distributed data were subjected to analysis of variance (ANOVA) at 5% probability. The means were compared by Tukey’s test (*p* < 0.05) when the ANOVA F-test detected interaction between diets and periods. As ultrasonographic data were non-parametric, they were analyzed by Wilcoxon’s test (*p* < 0.05), whereas cardiac and blood pressure data were analyzed by Student’s *t*-test (*p* < 0.05).

For the palatability test, 32 repetitions per test were considered (16 dogs × 2 days). Intake ratio data were analyzed by the paired Student’s *t*-test and the first choice by the Chi-square test, both at 5% probability.

## 3. Results

### 3.1. Digestibility and Fecal Characteristics

No vomiting, diarrhea, or coprophagia occurred, and all dogs remained healthy throughout the experimental period. There was no change in feed intake (*p* > 0.05) during the experiment (control = 207.9 ± 16.75 g and test = 204.6 ± 23.68 g on average of DM/animal/day). Similarly, the animals presented no weight variation throughout the study (mean initial weight: control = 13.69 ± 2.01 kg and test = 13.68 ± 2.11 kg; mean final weight: control = 13.40 ± 2.51 kg and test = 13.42 ± 2.60 kg). Furthermore, nutrients’ CTTAD, dietary ME, and fecal characteristics did not differ between treatments (*p* > 0.05; Table 2).

### 3.2. Blood Analysis

There were no differences in LDL, GGT, total protein, globulin, and albumin values between treatments (*p* > 0.05; Table 3). At the end of the experiment, dogs fed with the test diet showed lower concentrations of total cholesterol, triglycerides, and ALP when compared to animals in the control group (*p* < 0.05). In addition, on day 45, ALT had an interaction between diet and period, with a reduction only in the group fed the test diet (*p* < 0.05). Decreased HDL values and increased triglycerides, LDL, and VLDL values were observed in all dogs at the end of the experiment, regardless of the treatment (*p* < 0.05; Table 3).

Moreover, on day 45, the red blood cells count indicated decreased values of hemoglobin, hematocrit, mean corpuscular volume (MCV), mean corpuscular hemoglobin (MCH), and mean corpuscular hemoglobin concentration (MCHC, *p* < 0.05), as well as increased monocytes, regardless of diet (*p* < 0.05; Table 3).

### 3.3. Abdominal Ultrasound, Echocardiogram, and Blood Pressure

There was no difference in the liver or splenic size, nor in the parenchymal organs’ echogenicity between diets and periods (*p* > 0.05; Table 4). However, moderate vascular changes—i.e., dilation of the hepatic vena cava and abdominal aorta (grade 1)—were observed in dogs fed the test diet (*p* < 0.05; Table 4).

There was no difference in the dog’s blood pressure (control = 170 mmHg and test = 174 mmHg) and heart rate (control = 128 bpm and test = 124 bpm) (*p* > 0.05).

### 3.4. Palatability

There was no difference in diets’ palatability. A 0.59 ± 0.162 intake ratio was observed for the control diet and 0.41 ± 0.162 for the test diet (*p* = 0.182). Furthermore, results obtained from the first choice analysis were 14 animals for control and 18 animals for test in a total of 32 observations (*p* = 0.872).

## 4. Discussion

Our results indicate that the evaluated herbal source of choline is a potential substitute for choline chloride in dog diets. The digestibility and fecal characteristics results showed no influence of the herbal source of choline on these variables. Similar results were found by Mendonza Martínez et al. [11] when evaluating the same herbal source of choline in dogs. The authors suggest that the contribution of choline-related compounds present in the herbal source, such as free choline, glycerophosphocholine, phosphocholine, phosphatidylcholine, and sphingomyelin, have been sufficient to contribute with dietary phospholipids to the various functions in digestion and nutrient absorption, especially lipids [11]. Therefore, the addition of choline from herbal sources does not alter the utilization of nutrients when compared to the standard one, as well as the fecal consistency, which remained at four, and is considered within the normal range [21].

The diet containing the herbal source of choline contributed to the reduction of serum ALP and ALT, although the levels remained within the normal range for dogs [22]. ALT is a hepato-specific enzyme in dogs located in the cytoplasm of hepatocytes and is released when there is light damage to cell membranes. Therefore, ALT is a good parameter for screening liver and heart diseases [23]. ALP, in turn, is not hepato-specific and may be increased when there is cholestasis, hepatic steatosis, or inflammation of the hepatic parenchyma. These diseases can obstruct small bile canaliculi and induce greater production and release of ALP [24].

There were also lower total cholesterol and serum triglycerides concentrations in dogs fed the diet containing the herbal source of choline at the end of the trial, in comparison to the control group. This lower concentration in serum cholesterol was also observed in dogs supplemented with increasing levels of the same herbal source of choline in the study of Mendoza-Martínez et al. [5]. In broilers, a reduction in cholesterol, triglycerides, and serum ALT was observed in animals fed an herbal source of choline [25]. This may be due to phosphatidylcholine, a phospholipid responsible for exporting triglycerides from the liver, being essential for synthesizing VLDL, which transports lipids to peripheral tissues [4].

Furthermore, dogs fed an herbal source of choline showed modulation of the expression of genes involved in the signaling pathway of peroxisome proliferator-activated receptors (PPAR) [5], which are important for modulating cholesterol and acting on dyslipidemia [26,27]. Without an adequate supply of choline for the synthesis of phosphatidylcholine, triglycerides are accumulated in the body, which can lead to liver disorders such as steatosis [28]. Thus, the decrease in serum cholesterol and triglycerides, as well as in ALT and ALP enzymes, indicate that the evaluated herbal source of choline may be beneficial for dogs’ health.

The higher serum levels of cholesterol and triglycerides presented at the end of the experiment were expected regardless of the alternative source of choline. This is due to the experimental diets having higher lipid content (approximately 16.8% AEE) in relation to the diet that the animals had been consuming before the beginning of the experiment (12.7% AEE). It is worth emphasizing that, despite this discrepancy, cholesterol and triglyceride values remained within the reference values for dogs [22].

The only apparently controversial result was the moderate dilation of large vessels, such as the hepatic vena cava and abdominal aorta, which was observed in animals fed the diet with the herbal source of choline. However, we observed no other hepatic or cardiac alterations that corroborate this result. No change in blood pressure was observed either. The ultrasound evaluation did not show changes in the size and echogenicity of organs such as the liver and pancreas, and it did not reveal abnormal fat accumulation or other problems in the liver. This result was corroborated by the biochemical and hematological data obtained in the study. We did not find any other studies reporting similar results in animals supplemented with choline. Furthermore, dogs fed for 60 days with an herbal source of choline (800 mg/kg of diet) showed a reduction in the expression of genes involved in the renin-angiotensin system and PPAR, which are related to the prevention of cardiovascular diseases [5]. Further long-term studies for health hazard evaluation are required.

The herbal source of choline contains many volatile compounds that are present in many plants, fruits, and flowers, which can stimulate the olfactory cells of animals [29]. Additionally, *Azadirachta indica*—one of the evaluated herbal choline compound plants—is a medicinal species notorious for its bitter taste [30], a characteristic that could interfere with the ingestion of the test diet. However, this effect was not observed. In fact, a previous study with dogs observed greater palatability of diets containing 200 and 400 mg/kg of herbal source of choline compared to the diet containing choline chloride [11]. For the researchers, this result showed the feasibility of replacing the conventional choline source with an alternative without negatively affecting dogs’ diet acceptability.

## 5. Conclusions

The use of 0.14% herbal source of choline can be a possible substitute for choline chloride in dog nutrition as it does not significantly alter nutrient digestibility, diet palatability, and fecal characteristics of the animals. Through its use it is possible to measure the improvement of some functions of lipid metabolism in dogs, mainly by the reduction of ALT and ALP enzymes, total cholesterol, and serum triglycerides. However, it is important that long-term studies validate these results.

## Figures and Tables

**Table 1:** Chemical composition of the experimental diets (dry matter basis).

Item	Unit	Control	Test
Dry matter	%	94.53	94.60
Crude protein	%	27.77	27.94
Crude fiber	%	3.85	3.37
Acid-hydrolyzed ether extract	%	16.89	16.75
Ash	%	7.13	7.27
Calcium	%	1.52	1.51
Phosphorus	%	1.06	1.09
Gross energy	Kcal/kg	5145.46	5120.51

**Table 2:** Means of coefficients of total tract apparent digestibility (CTTAD, %), metabolizable energy, and fecal characteristics of dogs fed diets containing choline chloride (control) or the herbal source of choline (test).

Item	Control	Test	SEM	*p*-Value
CTTAD, %				
Dry matter	77.1	77.6	0.486	0.668
Organic matter	80.5	81.0	0.424	0.565
Crude protein	82.4	83.4	0.346	0.172
Ether extract	90.1	90.2	0.236	0.708
Gross energy	81.5	82.5	0.443	0.556
Metabolizable energy, kcal/kg				
ME	4168.1	4194.2	24.91	0.617
Fecal Characteristics				
Dry matter, %	36.37	36.01	0.121	0.709
pH	6.56	6.60	0.068	0.761
Score	4	4	-	0.999

SEM: Standard error of the mean; *p*-value obtained by the Student’s *t*-test (*p* < 0.05); fecal score was analyzed by the Wilcoxon test (*p* < 0.05).

**Table 3:** Means of blood variables of dogs fed diets containing choline chloride (control) or the herbal source of choline (test) at the beginning and end of the experiment.

Item (Reference Value)	Control		Test		SEM	*p*-Value		
	Initial	Final	Initial	Final		Diet (D)	Period (P)	D × P
Biochemical profile								
Cholesterol (125–270 mg/dL)	172.55	215.50	157.95	170.07	7.69	0.042	0.062	0.271
HDL (33–140 mg/dL)	140.08	108.31	123.62	103.34	4.30	0.153	0.001	0.431
LDL (34–115 mg/dL)	16.71	93.25	19.06	68.24	7.74	0.264	<0.001	0.252
VLDL (6.5–16.9 mg/dL)	9.86	8.28	15.73	12.41	0.98	0.171	0.008	0.621
Triglycerides (<150 mg/dL)	46.66	63.81	41.35	50.06	2.57	0.042	0.006	0.342
ALT (10.0–102.0 U/L)	41.61 ^b^	50.17 ^a^	46.84 ^ab^	41.76 ^b^	1.78	0.373	0.342	0.019
ALP (20–150.0 U/L)	65.76	49.31	60.40	33.43	3.10	0.027	<0.001	0.263
Gamma–GT (0–18 U/L)	2.25	2.36	2.51	0.98	0.23	0.194	0.094	0.064
Total Protein (5.4–7.7 g/dL)	6.04	6.31	6.03	6.15	0.05	0.734	0.832	0.321
Globulin (2.3–5.2 g/dL)	2.63	2.74	2.59	2.63	0.04	0.421	0.392	0.672
Albumin (2.3–3.8 g/dL)	3.41	3.56	3.44	3.53	0.03	0.922	0.061	0.632
Erythogram								
Erythrocytes (5.5–8.5 mi/μL)	6.21	6.21	6.51	6.02	0.08	0.771	0.122	0.123
Hemoglobin (12–18 g/dL)	15.60	14.03	16.33	13.75	0.26	0.520	<0.001	0.152
Hematocrit (37–53%)	44.75	42.13	45.75	40.86	0.58	0.883	<0.001	0.211
MCV (60–77 u^3^)	72.23	67.94	70.57	67.96	0.61	0.482	0.007	0.472
MCH (19.5–24.5 pg)	25.22	22.62	25.82	22.87	0.35	0.373	<0.001	0.723
MCHC (30–36%)	34.87	33.29	35.68	33.70	0.31	0.82	0.004	0.712
Leukogram								
Leukocytes (6–17 × 10^3^/mm^3^)	12.71	12.80	11.91	11.22	0.40	0.083	0.522	0.442
Neutrophils (58–80%)	75.63	72.88	71.38	69.35	1.32	0.140	0.360	0.893
Lymphocytes (12–30%)	19.90	19.27	25.51	22.38	1.09	0.060	0.351	0.532
Monocytes (3-10%)	3.50	5.13	2.23	5.38	0.47	0.371	0.002	0.213
Platelets (150–800 × 10^3^/mm^3^)	328	395	397	373	14.35	0.393	0.442	0.114
Plasmatic Proteins (5.5–8.0 g/dL)	6.58	6.83	6.72	6.55	0.09	0.444	0.054	0.474

HDL: high-density lipoproteins; LDL: low-density lipoproteins; VLDL: very low-density lipoproteins; ALT: alanine aminotransferase; ALP: alkaline phosphatase; MCV: mean corpuscular volume; MCH: mean corpuscular hemoglobin; MCHC: mean corpuscular hemoglobin concentration; Gamma-GT: gamma-glutamyl transferase; SEM: standard error of the mean; ^a,b^ means followed by distinct lowercase letters differ by Tukey’s test with *p* < 0.05.

**Table 4:** Medians (1st; 3rd quartiles) of ultrasound variables of the abdominal region of dogs fed diets containing choline chloride (control) or an herbal source of choline (test).

Item	Control		*p*-Value	Test		*p*-Value	Final–Initial *		*p*-Value
	Initial	Final		Initial	Final		Control	Herbal source	
Liver size	0 (0;0.25)	0 (0;1.0)	1.000	0 (0;0.25)	0.5 (0;1.25)	0.248	0 (0;0.25)	0 (0;1.25)	0.727
Splenic size	0 (0;0.25)	0 (0;0.25)	1.000	0 (0;0.25)	0.5 (0;1.0)	0.282	0 (0;0)	0 (0;1.0)	0.174
Echogenicity of parenchymical organs	0 (0;0.25)	0.5 (0;0.25)	1.000	0 (0;1.0)	0.5 (0;1.0)	0.927	0 (0;0)	0 (0;0)	1.000
Vena cava dilation	0 (0;0)	0 (0;0)	1.000	0 (0;0)	0 (0.75;1.0)	0.015	0 (0;0)	1 (0.75;1.0)	0.015
Abdominal	0 (0;0)	0 (0;0)	1.000	0 (0;0)	1 (0.75;1.0)	0.015	0 (0;0)	1 (0.75;1.0)	0.015
aortic dilation									
Gallbladder	0 (0;0)	0 (0;0)	1.000	0 (0;0.25)	0 (0;1.0)	0.595	0 (0;0)	0 (0;0.25)	0.405
Stomach	0 (0;0)	0 (0;0)	1.000	0 (0;0.25)	0 (0;1.0)	0.182	0 (0;0)	0 (−0.25;1.0)	0.404
Pancreas	0 (0;0)	0 (0;1.0)	0.901	0 (0;1.0)	0 (0;1.0)	0.922	0 (0;1.0)	0 (−1.0;1.0)	0.688

* Final (day 45) minus initial (day 0) measurement of each animal; 0 = no changes in size and/or echogenicity; 1 = increase in size and/or echogenicity; and 2 = significant increase in size and/or echogenicity.

## Data Availability

All data are contained within the article.
